# POEMS syndrome (polyneuropathy, organomegaly, endocrinopathy, multiple myeloma and skin changes) with cranial vault plasmocytoma and the role of surgery in its management: a case report

**DOI:** 10.1186/1752-1947-7-245

**Published:** 2013-10-18

**Authors:** Julio Plata Bello, Victor Garcia-Marin

**Affiliations:** 1 Neuroscience Department, Hospital Universitario de Canarias, C/Ofra s/n. La Laguna, 38320, Santa Cruz de Tenerife, Spain

## Abstract

**Introduction:**

POEMS syndrome (an acronym of polyneuropathy, organomegaly, endocrinopathy, multiple myeloma and skin changes) is a paraneoplastic disorder related to an underlying plasma cell dyscrasia. The development of such a syndrome is rare and its association with calvarial plasmocytoma is even less common, with only two previous reported cases. We describe, in detail, an unusual presentation of cranial plasmocytoma associated with POEMS syndrome and briefly discuss the possible role of surgery in the management of this disease.

**Case presentation:**

We present the case of a 45-year-old Caucasian man who was admitted to our department presenting with progressive weakness in his lower limbs, enlarged lymph nodes and a large mass on the scalp with intense bone erosion. POEMS criteria were present and pathological studies confirmed a Castleman’s variant plasmocytoma. Clinical status improved noticeably after the excision of the plasmocytoma and the treatment was completed with radiotherapy and steroid pulse therapy.

**Conclusion:**

Cranial vault plasmocytoma and its association with POEMS syndrome are rare conditions with few previously reported cases. Although the role of surgery is not clearly defined in POEMS syndrome guidelines, the fact that there seems to be a better prognosis and clinical outcome when surgery is used as a part of the management in POEMS syndrome with cranial vault plasmocytoma is worth discussing.

## Introduction

POEMS syndrome is a paraneoplastic disorder related to an underlying plasma cell dyscrasia. The term POEMS is an acronym encompassing the features of the syndrome: polyneuropathy, organomegaly, endocrinopathy, multiple myeloma and skin changes (Table 1) [1,2]. The term for this rare syndrome was first coined by Bardwick *et al*. in 1980 [3] and its real incidence and prevalence is unknown.

**Table 1:** Criteria for the diagnosis of POEMS syndrome

Mandatory major criteria	Polyneuropathy
	Monoclonal plasmaproliferative disorder
Other major criteria	Castleman’s disease
	Sclerotic bone lesions
	Elevated serum or plasma vascular endothelial growth factor (VEGF) levels
Minor criteria	Organomegaly (splenomegaly, hepatomegaly, or lymphadenopathy)
	Extravascular volume overload (peripheral edema, ascites or pleural effusion)
	Endocrinopathy (adrenal, thyroid, pituitary, gonadal, parathyroid, pancreatic)
	Skin changes (hyperpigmentation, hypertrichosis, plethora, hemangiomata, white nails)
	Papilledema
	Thrombocytosis or polycythemia

The existence of both mandatory major criteria and at least one of the other major criteria and one of the minor criteria are necessary for the diagnosis [4].

We report the case of a patient who suffered a POEMS syndrome associated with a cranial vault plasmocytoma managed with surgery, radiotherapy and steroids.

## Case presentation

A 45-year-old Caucasian man was admitted to the Accident and Emergency Department of our hospital after presenting with a two-week history of progressive walk impairment associated with paresthesias around his mouth and the fingers on his right hand. The most remarkable features of his medical history were the presence of type 2 diabetes (recently diagnosed), dislipemia, high blood pressure and morbid obesity (body mass index: 41.3).

A medical examination showed a lower limb paresis (grade 3/5), which was more prominent in dorsal flexion of both feet, an absence of reflexes and a loss of superficial sensitivity in his left limb. The presence of enlarged lymph nodes in the cervical area was also evident; they were variable in size (some of them of more than 1cm) and mobile. Furthermore, a large scalp mass was identified in the parietal-occipital area; it was stuck to the skull, had an irregular shape, a tough texture, and palpation was not painful. Apart from these findings, the presence of a bilateral gynecomastia and a hyperpigmentation of the skin, mostly of the areoles, were noteworthy.

An electromyography (EMG) test was performed to better determine the neurological symptoms. The EMG showed a motor-dominant polyneuropathy with demyelinating features in his lower limbs. The administration of steroids only partially improved his neurological symptoms.

**Figure 1:**
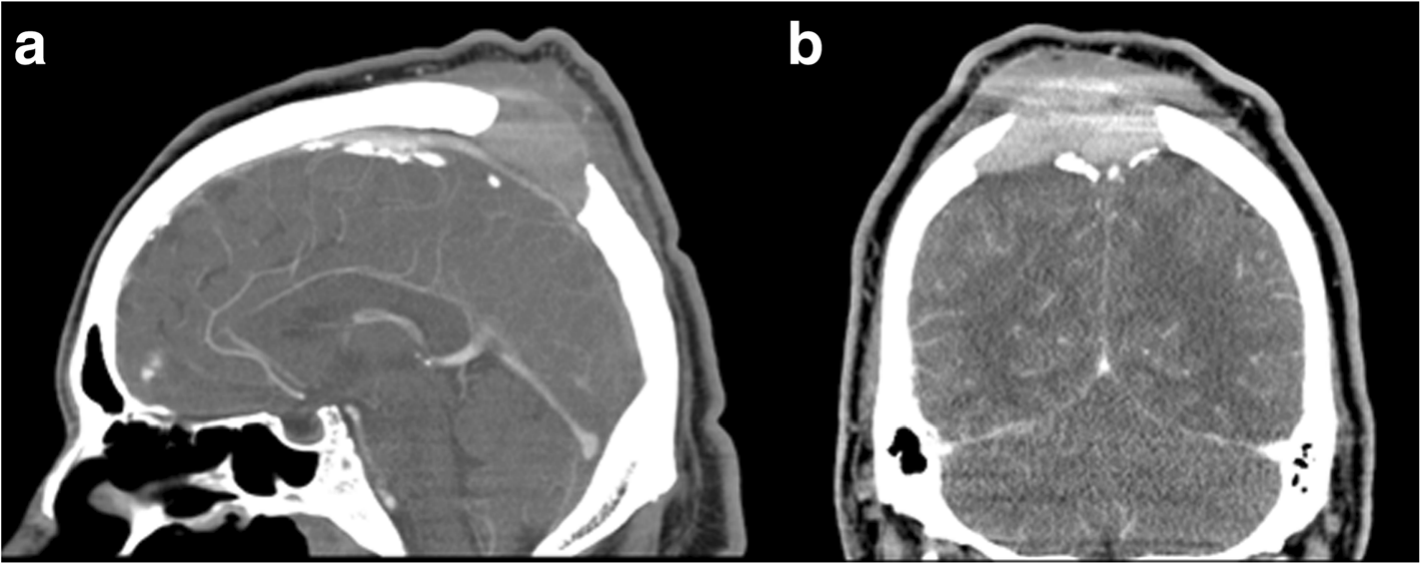
**Head computed tomography scan. (a)** Sagittal and **(b)** frontal views of a contrast-enhanced cranial computed tomography scan. The intense bone destruction above the sagittal sinus can be seen. No brain infiltration seems to be present.

A cranial computed tomography (CT) scan was performed and showed a mass in the scalp with intense underlying bone erosion (Figure 1). The mass was in contact with the posterior part of the sagittal sinus, but did not invade it. There were no signs of brain damage or infiltration and the tumor presented intense vascularization, mainly from vessels of the external carotid artery (ECA). A magnetic resonance imaging (MRI) scan could not be performed because of our patient’s high body weight. A whole-body CT scan was carried out and revealed adenopathies in the cervical area, mediastinum and retroperitoneum. Splenomegaly was also identified.

**Figure 2:**
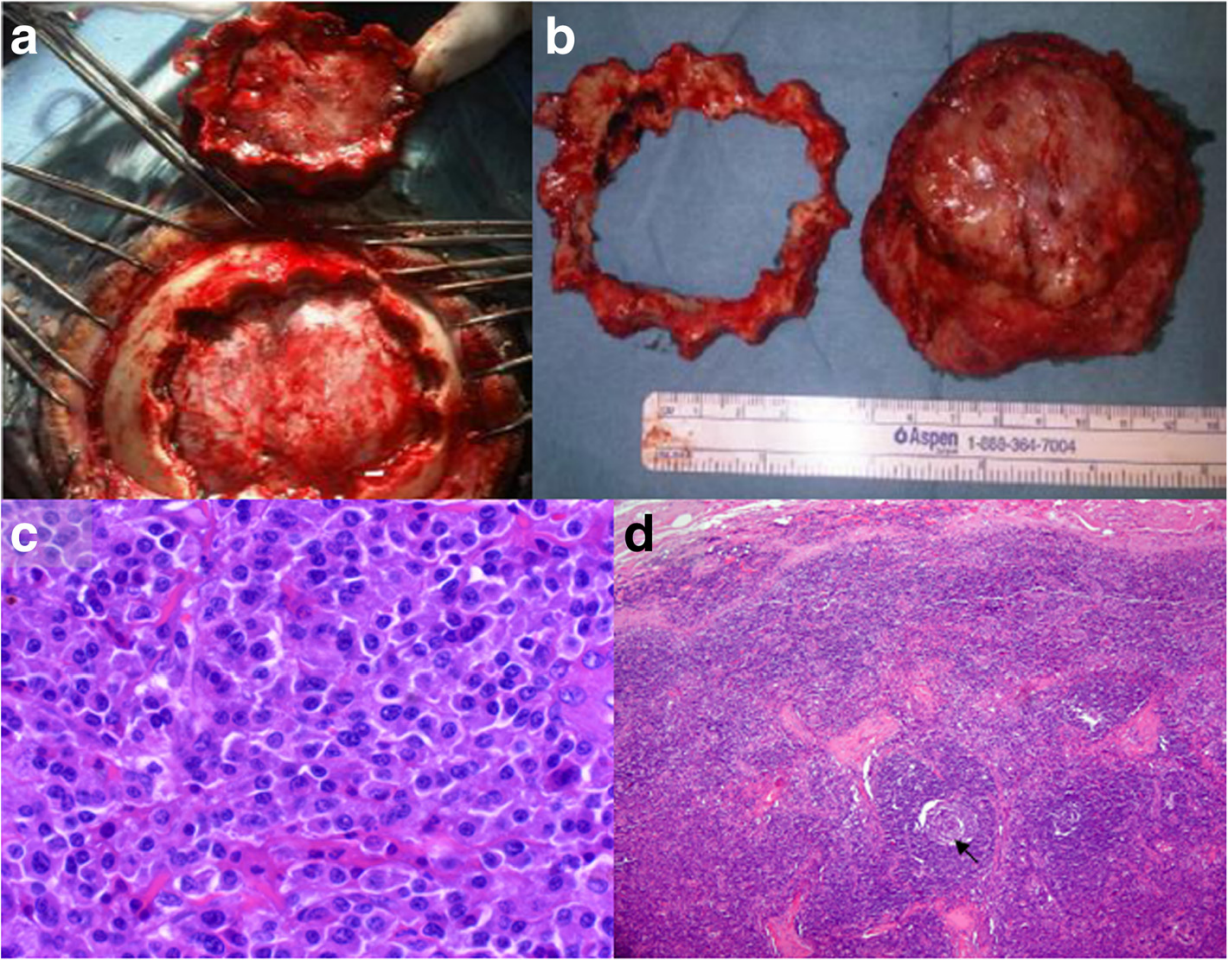
**Operative and pathological views. (a)** and **(b)** show the circular craniectomy and the complete excision of the mass. Observe that the dura mater does not seem to be infiltrated or damaged; **(c)** hematoxilin and eosin preparation from the mass showing multiple plasmatic cell infiltration; **(d)** hematoxilin and eosin preparation from the lymph node biopsy with peripheral follicular proliferation and hyalinization of the vessels (black arrow) and the presence of plasma cells. These are the typical findings of Castleman’s disease.

His laboratory test results showed a significant increase of immunoglobulin G (IgG) with a clear peak in the proteinogram, increase of lambda and kappa light chains in serum and high levels of vascular endothelial growth factor (VEGF) (Table 2). No Bence Jones proteinuria was identified. A bone marrow biopsy showed reactive changes and intense plasmocytosis and pathological analysis of one of the cervical lymph nodes showed reactive changes with peripheral follicular proliferation, hyalinization of the vessels and the presence of plasma cells (Figure 2d). These findings were compatible with Castleman’s disease. Serological tests for human herpes virus-8 (HHV-8) and human immunodeficiency virus (HIV) infection were performed, with negative results, because of the association between Castleman’s disease and these viral infections [5,6]. Castleman’s disease occurs in about 11 to 30% of patients with POEMS syndrome [7].

**Table 2:** Laboratory test results at our patient’s initial assessment

**Blood cell count**		
Red blood cell count	4.96×10E6/mm3	(4.50–5.90)
White blood cell count	7.5×10E3/mm3	(4.5–11.1)
Neutrophils	4.41×10E3/mm3	(1.70–7.00)
Lymphocytes	2.04×10E3/mm3	(1.00–4.80)
Monocytes	0.73×10E3/mm3	(0.10–0.80)
Eosinophils	0.24×10E3/mm3	(0.00–0.40)
Basophils	0.07×10E3/mm3	(0.00–0.20)
Platelets	510×10E3/mm3	(150–400)
Erythrocyte sedimentation rate	53mm/hour	(2–14)
**Proteins**		
Immunoglobulin G (IgG)	3473mg/dL	(800–1.800)
Immunoglobulin A (IgA)	285mg/dL	(90–450)
Immunoglobulin M (IgM)	93mg/dL	(65–265)
Kappa light chain (plasma)	824.00mg/dL	(170.00–370.00)
Lambda light chain (plasma)	433.00mg/dL	(90.00–210.00)
Kappa/Lambda (plasma)	1.90	(1.42–2.63)
Vascular endothelial growth factor (VEGF)	344.00pg/mL	(20–65)
Immunofixation	Lambda light chains are observed; alternatively, a homogeneous IgG band is shown.	
**Tumoral markers**		
Beta-2 microglobulin	2.37mg/L	(0.69–3.40)
CA 72.4	0.98U/mL	(0.00–6.00)
CA 15.3	11.9U/mL	<31.5
CA 19.9	6.7U/mL	(0.0–60.0)
Carcinoembryonic antigen (CEA)	<0.5ng/mL	<5.2
**Hormones**		
B12 vitamin	243.0pg/mL	(179.0–1,160.0)
Folic acid	2.3ng/mL	(3.0–17.0)
Antithyroglobulin	37.40U.I./mL	(0.00–40.00)
Thyroid-stimulating hormone (TSH) (basal)	1.7600μU/mL	(0.4000–4.0000)
Thyroxine (T4) (free)	1.20ng/dL	(0.70 - 1.85)

The decision was taken to surgically remove the parietal-occipital mass, with a previous embolization of the main arteries feeding the tumor in order to avoid excessive bleeding during surgery. The patient was operated on under general anesthesia. We performed a circular craniectomy around the tumor and a complete excision of the lesion by dissecting its adherences with the dura mater, which did not seem to be damaged (Figure 2a and 2b). A titanium mesh was placed in the osseous defect.

Pathological analyses concluded that the tumor was a well-differentiated plasmocytoma with expression of CD38, IgG and lambda light chain (Figure 2c). A bone marrow biopsy showed reactive changes with intense plamocytosis.

Subsequently, according to the definitive pathological results, dexamethasone pulse therapy was initiated (three pulses per month of 20mg/day for four days) and the patient also received radiotherapy to the skull to complete the treatment, with an accumulated dose of 50Gy in the surgical field.

As mentioned above, his neurological status improved slightly with the initial prescription of steroids, but neurological deficits recovered progressively after surgery. The rest of the POEMS features also improved after surgery and even more so when steroids and radiotherapy were initiated (two weeks after surgery).

**Figure 3:**
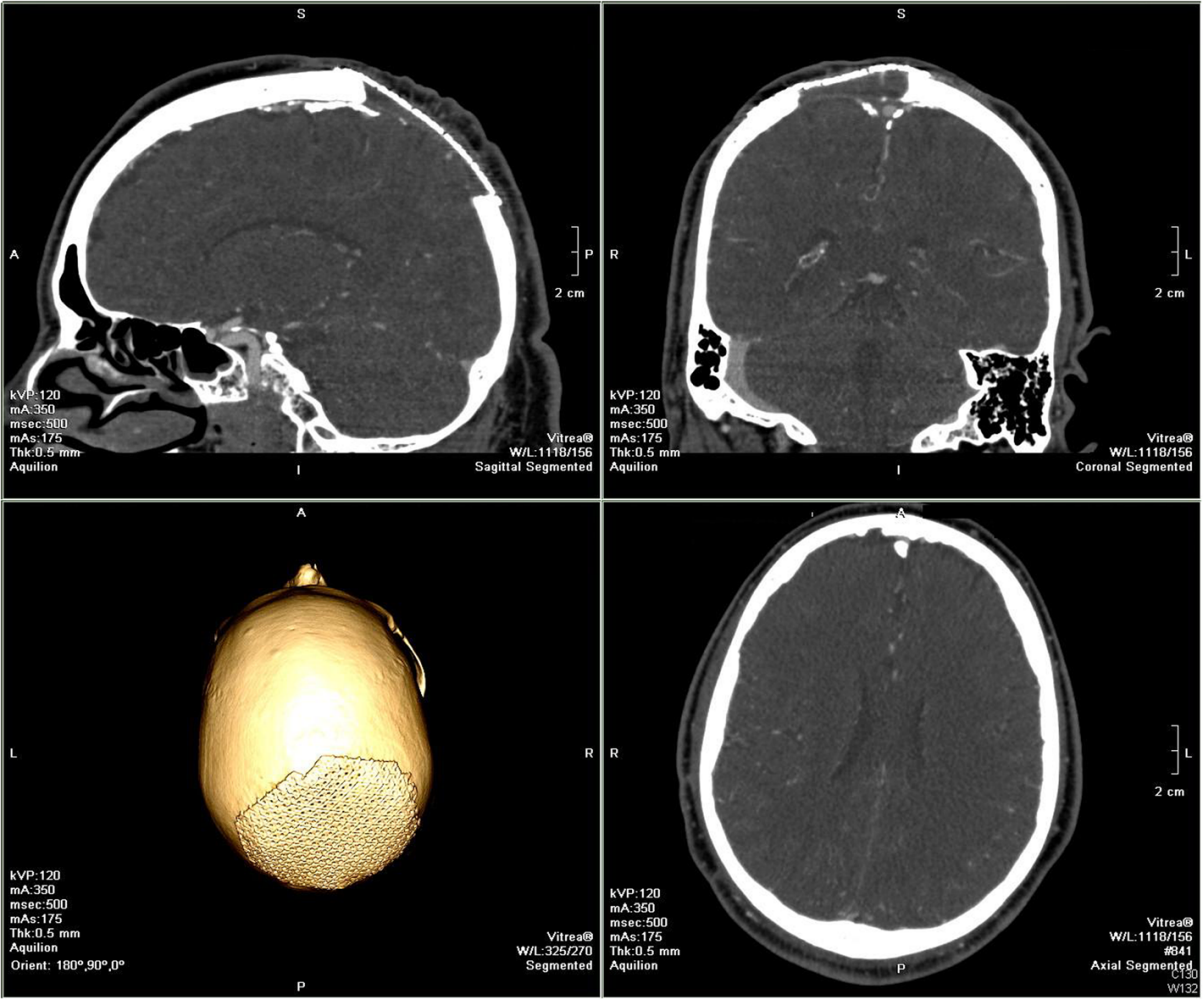
**Six-month follow-up computed tomography scan with three-dimensional reconstruction.** The cranial defect is shown covered by a titanium mesh. No signs of a local relapse of plasmotcytoma are evident.

Apart from his clinical status, his response to treatment was evaluated with positron emission tomography (PET), which was performed two months after the last steroid pulse. It showed a complete resolution of adenopathies in the different areas and no abnormal activity was identified in the skull. Moreover, vascular endothelial growth factor (VEGF) and lambda chain serum levels normalized progressively. During the 18-month follow-up, there have not been any clinical or laboratory abnormalities to indicate a recurrence of the disease. No complications related to the surgical procedure were reported (Figure 3).

## Discussion

We report a rare case of a Castleman’s disease variant of POEMS syndrome with a cranial plasmocytoma with a noticeable clinical improvement after surgical excision of the cranial tumor.

Differentiating POEMS syndrome from other plasma cell disorders (for example multiple myeloma) is based on the specific combination of multi-systemic symptoms, lambda chain- restricted M protein and high serum VEGF levels [7]. Although the reported case presented not only high levels of lambda chain in serum but also of kappa light chain in serum (Table 2), the high levels of VEGF as well as that established by the diagnosis criteria (Table 1) led to the final diagnosis of POEMS syndrome. The high specificity of VEGF should be mentioned when differentiating among POEMS syndrome, multiple myeloma, amyloidosis, monoclonal gammapathy and chronic inflammatory demyelinating polyradiculoneuropathy (CIDP). While this factor shows selectively increased serum levels in POEMS syndrome, the other entities usually present normal levels [7].

The skull is an uncommon location for such hematological lesions, and the frequency is even lower when only the cranial vault is considered. In 2000, Khouja *et al.* reviewed the reported cases of plasmocytomas with cranial vault location. At that time, 36 cases had been published [8] and in the last 12 years different authors have reported five more cases. Therefore, a total of 41 cases of cranial vault plasmocytoma have been reported most of which were located in the parietal and occipital bones; frontal localization seems to be less frequent. Not all cases had erosion of the underlying bone and most of them showed an improvement of the symptoms after surgical removal, which is generally followed by radiotherapy [8,9].

The association of POEMS syndrome and cranial vault plasmocytoma is even more uncommon than skull plasmocytoma. To date, there are only two reported cases presenting with such a cranial lesion associated with POEMS syndrome [10,11]. One of them also presented with an osteolytic lesion in the parietal-occipital area, which was treated with surgery and radiation therapy [11]. The other reported case did not show bone erosion and the patient developed an immune complex vasculitis [10]. In both cases, a clinical improvement was reported after surgical excision of the plasmocytoma. Surgery may be also recommended in cranial plasmocytomas when the bone defect is large enough to consider it necessary to reconstruct the cranial vault [12,13].

However, neither Dispenzieri (2012) in a review about the management of POEMS syndrome nor Li and Zhou (2013) in another review about the new advances in the diagnosis and treatment of POEMS syndrome included surgery as a therapeutic element [7,14]. Differences in the management of the plasmocytoma depending on its location were not specified by the above-mentioned authors and, as we have described, the cranial vault seems to be a special location where surgery might have a certain role to play.

Therefore, POEMS syndrome with a cranial vault plasmocytoma might have a better prognosis and clinical outcome if surgery is included in the management of the disease. Needless to say, some case reports do not provide a great deal of evidence about the role of surgery in this disease, but the low frequency of skull plasmocytomas with POEMS syndrome and the outcome of the reported cases suggest that surgery should be taken into consideration in the global management of the disease.

## Conclusions

We report a new case of a POEMS syndrome associated with a cranial vault plasmocytoma. Adding surgery to the management of this disease might improve clinical outcome and prognosis. More case series are necessary to have a greater level of evidence about the proper management of these patients and, especially, the role played by surgery.

## Consent

Written informed consent was obtained from the patient for publication of this case report and any accompanying images. A copy of the written consent is available for review by the Editor-in-Chief of this journal.

## Competing interests

The authors declare that they have no competing interests.

## Authors’ contributions

PJ and GMV analyzed and interpreted the patient data regarding the diagnosis and performed the surgery. PJ was the major contributor in writing the manuscript. Both authors read and approved the final manuscript.
